# 2,5-Bis{2,2-bis­[4-(dimethyl­amino)­phen­yl]ethen­yl}-*N*,*N*′-diphenyl-*N*,*N*′-dipropyl­benzene-1,4-diamine

**DOI:** 10.1107/S160053681100910X

**Published:** 2011-03-12

**Authors:** Volker Schmitt, Dieter Schollmeyer, Heiner Detert

**Affiliations:** a University Mainz, Duesbergweg 10-14, 55099 Mainz, Germany

## Abstract

The title compound, C_60_H_68_N_6_, was prepared by Horner olefination of a terephthaldialdehyde and a diaryl­methyl phospho­nate. There is one half-mol­ecule, located on an inversion centre, in the asymmetric unit. The dihedral angle between the plane of the vinyl­ene unit and the central ring is 36.79 (15)°, while those between the vinyl­ene unit and the lateral phenyl rings are 53.04 (10) and 53.74 (9)°.

## Related literature

For conjugated oligomers with basic sites as sensing materials for polarity and cations, see: Detert & Sugiono (2004 ▶, 2005 ▶); Wilson & Bunz (2005 ▶); Zucchero *et al.* (2009 ▶). For typical synthetic approaches to larger stilbenoid dyes, see: Drefahl & Plötner (1961 ▶); Stalmach *et al.* (1996 ▶). For crystal structures of phenyl­ene­vinyl­ene oligomers, see: van Hutten *et al.* (1999 ▶); Detert *et al.* (2001 ▶). For optical properties of dyes which are highly sensitive towards environmental changes, see: Detert *et al.* (2001 ▶); Strehmel *et al.* (2003 ▶); Nemkovich *et al.* (2010 ▶). For the synthesis of the title compound, see: Schmitt (2005 ▶); Zheng *et al.* (2003 ▶).
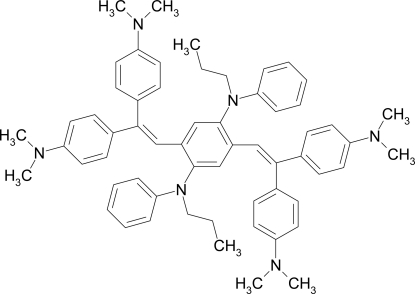


## Experimental

### 

#### Crystal data

C_60_H_68_N_6_
*M* *_r_* = 873.20
Monoclinic, 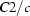

*a* = 20.485 (9) Å
*b* = 12.0782 (16) Å
*c* = 21.108 (9) Å
β = 107.60 (2)°
*V* = 4978 (3) Å^3^
*Z* = 4
Cu *K*α radiation
μ = 0.52 mm^−1^
*T* = 193 K
0.50 × 0.30 × 0.20 mm

#### Data collection

Enraf–Nonius CAD-4 diffractometer
4859 measured reflections
4720 independent reflections
3352 reflections with *I* > 2σ(*I*)
*R*_int_ = 0.064
3 standard reflections every 60 min  intensity decay: 2%

#### Refinement

*R*[*F* ^2^ > 2σ(*F* ^2^)] = 0.074
*wR*(*F* ^2^) = 0.232
*S* = 1.09
4720 reflections
303 parameters
H-atom parameters constrained
Δρ_max_ = 0.28 e Å^−3^
Δρ_min_ = −0.22 e Å^−3^

### 

Data collection: *CAD-4 Software* (Enraf–Nonius, 1989 ▶); cell refinement: *CAD-4 Software*; data reduction: *CORINC* (Dräger & Gattow, 1971 ▶); program(s) used to solve structure: *SIR97* (Altomare *et al.*, 1999 ▶); program(s) used to refine structure: *SHELXL97* (Sheldrick, 2008 ▶); molecular graphics: *PLATON* (Spek, 2009 ▶); software used to prepare material for publication: *PLATON*.

## Supplementary Material

Crystal structure: contains datablocks I, global. DOI: 10.1107/S160053681100910X/bt5488sup1.cif

Structure factors: contains datablocks I. DOI: 10.1107/S160053681100910X/bt5488Isup2.hkl

Additional supplementary materials:  crystallographic information; 3D view; checkCIF report

## Figures and Tables

**Fig. 1.**
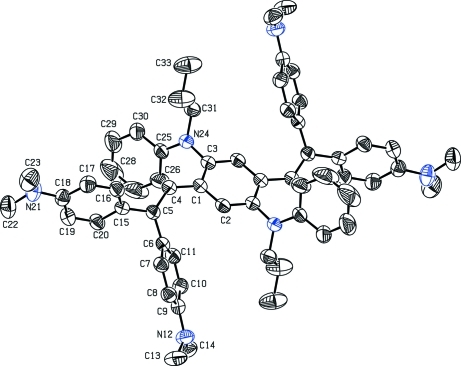
View of the title compound. Displacement ellipsoids are drawn at the 50% probability level.

## supplementary crystallographic information

### Comment

The title compound was prepared as part of a project focusing on chromophores and fluorophores based on oligo(phenylenevinylene)s with multiple basic sites, see: Detert & Sugiono (2004, 2005). The optical properties of these dyes are highly sensitive towards changes of the environment see: Detert *et al.* (2001); Strehmel *et al.* (2003) and Nemkovich *et al.* (2010).

The compound, prepared in a twofold Horner olefination of a central dialdehyde and a diarylmethylphosphonate, crystallized from chloroform/methanol in block-shaped crystals. The packing of the molecules is based on van-der-Waals interactions. The molecules contain a center of symmetry, due to sterical crowding, the rigid units phenylene and vinylene show large torsion angles disturbing the conjugation along the π-system. The torsion angle C2—C1—C4—C5 amount to -33.7 (4)° between the central ring and the vinylene units and to 49° - 55° between vinylene and lateral phenyl rings. These subunits are essentially planar, with torsion angles of less than 3° in the phenylene rings and a maximum distortion of -6.4 (4) along the *cis*-configurated C1—C4—C5—C6 vinylene bond. The geometries of the central and peripheral amino groups are significantly different due to the different substitution: diarylalkylamine *versus* aryldialkylamine, and the sterical crowding in the middle of the molecule. The C3—N24-bonds of the *p*-aminoaniline moiety 1.423 (3) Å are significantly longer than all other aryl-N bonds: C18—N21: 1.387 (3) Å; C9—N12: N24—C25: 1.394 (3) and the peripheral nitrogen atoms are slightly planarized with sums of the C—N bond angles of 353.6° around N12 and 355.4° around N21 but the sum of the bond angles at the *p*-aminoaniline N atoms amount to 359.9°. Dihedral angles of the disubstituted amino groups and the mean planes of the adjacent phenylene ring are small for the dimethylamino groups (C13—N12—C14)-(C6 - C11): 25.8 (3)° and (C22—N21—C23)-(C15 - C20): 22.4 (3)° but, large for the *p*-aminoaniline unit (C25—N24—C31)-(C2—C3—C1): 59.3 (3)°.

### Experimental

The title compound was prepared *via* Horner olefination of a solution of 2,5-Bis(*N*-propyl-*N*-phenylamino)terephthalaldehyde (120 mg, 0.30 mmol) (Schmitt, 2005) and diethyl bis[4-(*N*,*N*-dimethylamino)phenyl]methylphosphonate (Zheng *et al.*, 2003) (257 mg, 0.66 mmol) in anhydrous THF (30 ml) and potassium-*t*-butylate (112 mg, 0.99 mmol) at 273 K. After stirring for 30 min, the mixture was allowed to reach ambient temperature and after 4 h stirring, water (60 ml) was added and the product extracted with ethyl acetate (3 *x* 30 ml). The pooled organic layers were washed with brine, dried over MgSO_4_ and concentrated. The residue was purified by chromatography and recrystallization from dichloromethane/methanol. Yield: 190 mg (50%) of an orange solid with m.p. = 487 K.

### Refinement

Hydrogen atoms attached to carbons were placed at calculated positions with C—H = 0.95 Å (aromatic) or 0.98–0.99 Å (*sp*^3^ C-atom). All H atoms were refined in the riding-model approximation with isotropic displacement parameters (set at 1.2–1.5 times of the *U*_eq_ of the parent atom).

### Crystal data

**Table:**

C_60_H_68_N_6_	*F*(000) = 1880
*M**_r_* = 873.20	*D*_x_ = 1.165 Mg m^−^^3^
Monoclinic, *C*2/*c*	Melting point: 487 K
Hall symbol: -C 2yc	Cu *K*α radiation, λ = 1.54178 Å
*a* = 20.485 (9) Å	Cell parameters from 25 reflections
*b* = 12.0782 (16) Å	θ = 25–42°
*c* = 21.108 (9) Å	µ = 0.52 mm^−^^1^
β = 107.60 (2)°	*T* = 193 K
*V* = 4978 (3) Å^3^	Block, orange
*Z* = 4	0.50 × 0.30 × 0.20 mm

### Data collection

**Table:**

Enraf–Nonius CAD-4 diffractometer	*R*_int_ = 0.064
Radiation source: rotating anode	θ_max_ = 69.9°, θ_min_ = 4.3°
graphite	*h* = 0→24
ω/2θ scans	*k* = −14→0
4859 measured reflections	*l* = −25→24
4720 independent reflections	3 standard reflections every 60 min
3352 reflections with *I* > 2σ(*I*)	intensity decay: 2%

### Refinement

**Table:**

Refinement on *F*^2^	Primary atom site location: structure-invariant direct methods
Least-squares matrix: full	Secondary atom site location: difference Fourier map
*R*[*F*^2^ > 2σ(*F*^2^)] = 0.074	Hydrogen site location: inferred from neighbouring sites
*wR*(*F*^2^) = 0.232	H-atom parameters constrained
*S* = 1.09	*w* = 1/[σ^2^(*F*_o_^2^) + (0.1466*P*)^2^ + 0.3409*P*] where *P* = (*F*_o_^2^ + 2*F*_c_^2^)/3
4720 reflections	(Δ/σ)_max_ < 0.001
303 parameters	Δρ_max_ = 0.28 e Å^−^^3^
0 restraints	Δρ_min_ = −0.22 e Å^−^^3^

### Special details

**Table:**

Experimental. ^1^H-NMR (CDCl_3_, 400 MHz): δ (ppm) = 7.24 (s, 2H), 7.10 (t, 8H), 6.87 (d, ^3^J = 8.7 Hz, 4H), 6.83 (t, 4H), 6.78 (d, ^3^J = 8.2 Hz, 8H), 6.76 (s, 2H), 6.67 (d, ^3^J = 8.6 Hz, 4H), 6.50 (s, 2H), 6.48 (d, ^3^J = 8.7 Hz, 4H), 6.43 (d, ^3^J = 8.6 Hz, 4H), 2.89 (s, 24H). ^13^C-NMR (CDCl_3_, 75 MHz): δ (ppm) = 149.6, 149.3, 147.5, 143.3, 142.3, 136.9, 132.2, 131.0, 128.7, 121.8, 121.0, 120.4 , 112.0, 111.6, 40.4, 20.4, 11.5. UV-vis (CH_2_Cl_2_): λ_max_ = 406 nm, ε = 33680 cm^2^/mmol.
Geometry. All e.s.d.'s (except the e.s.d. in the dihedral angle between two l.s. planes) are estimated using the full covariance matrix. The cell e.s.d.'s are taken into account individually in the estimation of e.s.d.'s in distances, angles and torsion angles; correlations between e.s.d.'s in cell parameters are only used when they are defined by crystal symmetry. An approximate (isotropic) treatment of cell e.s.d.'s is used for estimating e.s.d.'s involving l.s. planes.
Refinement. Refinement of *F*^2^ against ALL reflections. The weighted *R*-factor *wR* and goodness of fit *S* are based on *F*^2^, conventional *R*-factors *R* are based on *F*, with *F* set to zero for negative *F*^2^. The threshold expression of *F*^2^ > σ(*F*^2^) is used only for calculating *R*-factors(gt) *etc*. and is not relevant to the choice of reflections for refinement. *R*-factors based on *F*^2^ are statistically about twice as large as those based on *F*, and *R*- factors based on ALL data will be even larger.

### Fractional atomic coordinates and isotropic or equivalent isotropic displacement parameters (Å^2^)

**Table:**

	*x*	*y*	*z*	*U*_iso_*/*U*_eq_	
C1	0.21619 (12)	0.6465 (2)	0.48219 (12)	0.0467 (6)	
C2	0.28093 (12)	0.6668 (2)	0.47567 (12)	0.0483 (6)	
H2	0.3025	0.6096	0.4584	0.058*	
C3	0.18502 (12)	0.7339 (2)	0.50685 (12)	0.0462 (6)	
C4	0.18219 (12)	0.5391 (2)	0.46690 (12)	0.0469 (6)	
H4	0.1543	0.5189	0.4937	0.056*	
C5	0.18481 (12)	0.4645 (2)	0.41993 (11)	0.0440 (5)	
C6	0.21911 (11)	0.4822 (2)	0.36801 (11)	0.0430 (5)	
C7	0.26348 (12)	0.4027 (2)	0.35679 (12)	0.0463 (6)	
H7	0.2729	0.3386	0.3841	0.056*	
C8	0.29436 (13)	0.4136 (2)	0.30741 (13)	0.0500 (6)	
H8	0.3254	0.3583	0.3025	0.060*	
C9	0.28079 (12)	0.5045 (2)	0.26460 (12)	0.0475 (6)	
C10	0.23708 (13)	0.5856 (2)	0.27643 (13)	0.0485 (6)	
H10	0.2276	0.6498	0.2492	0.058*	
C11	0.20742 (13)	0.5744 (2)	0.32659 (12)	0.0490 (6)	
H11	0.1781	0.6314	0.3331	0.059*	
N12	0.30968 (12)	0.5151 (2)	0.21328 (11)	0.0596 (6)	
C13	0.34078 (19)	0.4193 (3)	0.19338 (18)	0.0781 (10)	
H13A	0.3765	0.3900	0.2318	0.117*	
H13B	0.3611	0.4405	0.1587	0.117*	
H13C	0.3058	0.3624	0.1761	0.117*	
C14	0.28180 (17)	0.5965 (3)	0.16197 (16)	0.0688 (8)	
H14A	0.2329	0.5822	0.1412	0.103*	
H14B	0.3057	0.5919	0.1283	0.103*	
H14C	0.2879	0.6707	0.1817	0.103*	
C15	0.14780 (12)	0.3576 (2)	0.41640 (11)	0.0442 (5)	
C16	0.15885 (13)	0.2878 (2)	0.47074 (12)	0.0498 (6)	
H16	0.1926	0.3070	0.5111	0.060*	
C17	0.12212 (14)	0.1909 (2)	0.46776 (14)	0.0549 (7)	
H17	0.1316	0.1446	0.5059	0.066*	
C18	0.07141 (12)	0.1598 (2)	0.40984 (14)	0.0510 (6)	
C19	0.06118 (13)	0.2285 (2)	0.35475 (14)	0.0542 (6)	
H19	0.0276	0.2093	0.3142	0.065*	
C20	0.09921 (14)	0.3244 (2)	0.35803 (13)	0.0512 (6)	
H20	0.0918	0.3686	0.3193	0.061*	
N21	0.03150 (13)	0.0666 (2)	0.40884 (14)	0.0673 (7)	
C22	−0.01593 (18)	0.0313 (3)	0.34656 (19)	0.0805 (10)	
H22A	−0.0489	0.0907	0.3285	0.121*	
H22B	−0.0404	−0.0349	0.3539	0.121*	
H22C	0.0093	0.0140	0.3151	0.121*	
C23	0.0580 (2)	−0.0200 (3)	0.4572 (2)	0.0881 (11)	
H23A	0.1007	−0.0488	0.4518	0.132*	
H23B	0.0243	−0.0799	0.4503	0.132*	
H23C	0.0668	0.0102	0.5021	0.132*	
N24	0.11885 (10)	0.7210 (2)	0.51472 (10)	0.0497 (5)	
C25	0.06185 (12)	0.6998 (2)	0.46013 (12)	0.0486 (6)	
C26	0.06600 (15)	0.7106 (3)	0.39625 (14)	0.0683 (9)	
H26	0.1082	0.7313	0.3897	0.082*	
C27	0.0099 (2)	0.6919 (5)	0.34216 (18)	0.1160 (19)	
H27	0.0141	0.6985	0.2987	0.139*	
C28	−0.0521 (2)	0.6637 (5)	0.3498 (2)	0.124 (2)	
H28	−0.0910	0.6514	0.3122	0.149*	
C29	−0.05672 (18)	0.6539 (4)	0.4124 (2)	0.0959 (14)	
H29	−0.0996	0.6355	0.4183	0.115*	
C30	−0.00108 (14)	0.6698 (3)	0.46759 (16)	0.0609 (7)	
H30	−0.0055	0.6604	0.5108	0.073*	
C31	0.11212 (13)	0.7389 (2)	0.58080 (13)	0.0540 (6)	
H31A	0.0648	0.7633	0.5765	0.065*	
H31B	0.1438	0.7987	0.6033	0.065*	
C32	0.1277 (2)	0.6363 (4)	0.62248 (18)	0.0875 (12)	
H32A	0.1751	0.6121	0.6271	0.105*	
H32B	0.0962	0.5764	0.5999	0.105*	
C33	0.1201 (2)	0.6556 (4)	0.69168 (17)	0.0960 (14)	
H33A	0.1442	0.7236	0.7107	0.144*	
H33B	0.1398	0.5927	0.7205	0.144*	
H33C	0.0715	0.6627	0.6881	0.144*	

### Atomic displacement parameters (Å^2^)

**Table:**

	*U*^11^	*U*^22^	*U*^33^	*U*^12^	*U*^13^	*U*^23^
C1	0.0514 (12)	0.0491 (14)	0.0409 (12)	−0.0079 (10)	0.0160 (10)	−0.0105 (11)
C2	0.0514 (13)	0.0504 (15)	0.0440 (13)	−0.0024 (10)	0.0160 (10)	−0.0127 (11)
C3	0.0485 (12)	0.0505 (15)	0.0417 (12)	−0.0045 (10)	0.0167 (9)	−0.0093 (11)
C4	0.0519 (12)	0.0476 (14)	0.0434 (12)	−0.0059 (10)	0.0179 (10)	−0.0065 (11)
C5	0.0476 (11)	0.0452 (13)	0.0386 (12)	−0.0045 (10)	0.0123 (9)	−0.0020 (10)
C6	0.0475 (11)	0.0417 (13)	0.0393 (12)	−0.0044 (9)	0.0125 (9)	−0.0072 (10)
C7	0.0540 (13)	0.0385 (13)	0.0461 (13)	0.0005 (10)	0.0147 (10)	−0.0024 (10)
C8	0.0531 (13)	0.0461 (14)	0.0518 (14)	0.0028 (10)	0.0172 (11)	−0.0049 (11)
C9	0.0482 (12)	0.0469 (14)	0.0481 (13)	−0.0073 (10)	0.0154 (10)	−0.0057 (11)
C10	0.0548 (13)	0.0418 (14)	0.0499 (14)	−0.0016 (10)	0.0173 (11)	0.0023 (11)
C11	0.0542 (13)	0.0445 (14)	0.0483 (14)	0.0006 (10)	0.0155 (10)	−0.0051 (11)
N12	0.0667 (13)	0.0645 (16)	0.0563 (13)	0.0005 (11)	0.0316 (11)	0.0015 (12)
C13	0.089 (2)	0.089 (3)	0.075 (2)	0.0114 (19)	0.0523 (18)	0.0008 (19)
C14	0.0745 (18)	0.078 (2)	0.0602 (17)	−0.0070 (16)	0.0301 (14)	0.0106 (16)
C15	0.0479 (11)	0.0475 (14)	0.0386 (12)	−0.0022 (10)	0.0153 (9)	−0.0041 (10)
C16	0.0515 (13)	0.0520 (15)	0.0428 (13)	−0.0047 (11)	0.0094 (10)	−0.0026 (11)
C17	0.0586 (14)	0.0541 (16)	0.0501 (14)	−0.0031 (12)	0.0134 (11)	0.0099 (12)
C18	0.0475 (12)	0.0437 (14)	0.0620 (16)	0.0002 (10)	0.0168 (11)	−0.0017 (12)
C19	0.0531 (13)	0.0499 (16)	0.0526 (14)	−0.0022 (11)	0.0054 (11)	−0.0061 (12)
C20	0.0605 (14)	0.0484 (15)	0.0411 (13)	−0.0038 (11)	0.0098 (10)	−0.0009 (11)
N21	0.0619 (13)	0.0475 (14)	0.0880 (18)	−0.0083 (10)	0.0162 (12)	0.0049 (13)
C22	0.0738 (19)	0.059 (2)	0.103 (3)	−0.0172 (16)	0.0191 (18)	−0.0126 (19)
C23	0.101 (3)	0.054 (2)	0.113 (3)	−0.0063 (18)	0.037 (2)	0.015 (2)
N24	0.0468 (11)	0.0617 (14)	0.0425 (11)	−0.0074 (9)	0.0164 (8)	−0.0121 (10)
C25	0.0510 (13)	0.0464 (14)	0.0479 (14)	−0.0044 (10)	0.0142 (10)	−0.0128 (11)
C26	0.0606 (16)	0.097 (3)	0.0457 (15)	0.0038 (16)	0.0140 (12)	−0.0092 (16)
C27	0.086 (3)	0.207 (6)	0.0492 (19)	0.018 (3)	0.0109 (17)	−0.029 (3)
C28	0.064 (2)	0.198 (6)	0.089 (3)	0.005 (3)	−0.0083 (19)	−0.064 (3)
C29	0.0549 (17)	0.122 (4)	0.103 (3)	−0.0181 (19)	0.0137 (18)	−0.048 (3)
C30	0.0520 (14)	0.0620 (18)	0.0696 (18)	−0.0091 (12)	0.0199 (13)	−0.0150 (14)
C31	0.0545 (13)	0.0617 (17)	0.0475 (14)	−0.0002 (12)	0.0179 (10)	−0.0085 (13)
C32	0.099 (3)	0.099 (3)	0.075 (2)	0.044 (2)	0.0414 (19)	0.023 (2)
C33	0.089 (2)	0.144 (4)	0.061 (2)	0.044 (2)	0.0324 (17)	0.035 (2)

### Geometric parameters (Å, °)

**Table:**

C1—C2	1.396 (3)	C18—N21	1.387 (3)
C1—C3	1.412 (3)	C18—C19	1.392 (4)
C1—C4	1.461 (4)	C19—C20	1.386 (4)
C2—C3^i^	1.380 (4)	C19—H19	0.9500
C2—H2	0.9500	C20—H20	0.9500
C3—C2^i^	1.380 (4)	N21—C22	1.443 (4)
C3—N24	1.423 (3)	N21—C23	1.447 (4)
C4—C5	1.353 (3)	C22—H22A	0.9800
C4—H4	0.9500	C22—H22B	0.9800
C5—C6	1.485 (3)	C22—H22C	0.9800
C5—C15	1.487 (3)	C23—H23A	0.9800
C6—C7	1.391 (3)	C23—H23B	0.9800
C6—C11	1.391 (4)	C23—H23C	0.9800
C7—C8	1.380 (4)	N24—C25	1.394 (3)
C7—H7	0.9500	N24—C31	1.459 (3)
C8—C9	1.395 (4)	C25—C26	1.383 (4)
C8—H8	0.9500	C25—C30	1.393 (4)
C9—N12	1.388 (3)	C26—C27	1.372 (5)
C9—C10	1.399 (4)	C26—H26	0.9500
C10—C11	1.377 (4)	C27—C28	1.371 (7)
C10—H10	0.9500	C27—H27	0.9500
C11—H11	0.9500	C28—C29	1.359 (6)
N12—C13	1.443 (4)	C28—H28	0.9500
N12—C14	1.447 (4)	C29—C30	1.374 (4)
C13—H13A	0.9800	C29—H29	0.9500
C13—H13B	0.9800	C30—H30	0.9500
C13—H13C	0.9800	C31—C32	1.497 (5)
C14—H14A	0.9800	C31—H31A	0.9900
C14—H14B	0.9800	C31—H31B	0.9900
C14—H14C	0.9800	C32—C33	1.533 (5)
C15—C16	1.386 (4)	C32—H32A	0.9900
C15—C20	1.389 (3)	C32—H32B	0.9900
C16—C17	1.382 (4)	C33—H33A	0.9800
C16—H16	0.9500	C33—H33B	0.9800
C17—C18	1.395 (4)	C33—H33C	0.9800
C17—H17	0.9500		
			
C2—C1—C3	117.0 (2)	C20—C19—C18	121.1 (2)
C2—C1—C4	122.5 (2)	C20—C19—H19	119.5
C3—C1—C4	120.4 (2)	C18—C19—H19	119.5
C3^i^—C2—C1	123.0 (2)	C19—C20—C15	121.8 (2)
C3^i^—C2—H2	118.5	C19—C20—H20	119.1
C1—C2—H2	118.5	C15—C20—H20	119.1
C2^i^—C3—C1	120.0 (2)	C18—N21—C22	119.0 (3)
C2^i^—C3—N24	119.1 (2)	C18—N21—C23	118.8 (3)
C1—C3—N24	120.9 (2)	C22—N21—C23	115.7 (3)
C5—C4—C1	129.1 (2)	N21—C22—H22A	109.5
C5—C4—H4	115.4	N21—C22—H22B	109.5
C1—C4—H4	115.4	H22A—C22—H22B	109.5
C4—C5—C6	125.2 (2)	N21—C22—H22C	109.5
C4—C5—C15	118.8 (2)	H22A—C22—H22C	109.5
C6—C5—C15	115.8 (2)	H22B—C22—H22C	109.5
C7—C6—C11	116.3 (2)	N21—C23—H23A	109.5
C7—C6—C5	120.3 (2)	N21—C23—H23B	109.5
C11—C6—C5	123.3 (2)	H23A—C23—H23B	109.5
C8—C7—C6	122.3 (2)	N21—C23—H23C	109.5
C8—C7—H7	118.8	H23A—C23—H23C	109.5
C6—C7—H7	118.8	H23B—C23—H23C	109.5
C7—C8—C9	121.2 (2)	C25—N24—C3	120.86 (19)
C7—C8—H8	119.4	C25—N24—C31	121.3 (2)
C9—C8—H8	119.4	C3—N24—C31	117.7 (2)
N12—C9—C8	121.9 (2)	C26—C25—C30	117.8 (3)
N12—C9—C10	121.4 (2)	C26—C25—N24	120.4 (2)
C8—C9—C10	116.6 (2)	C30—C25—N24	121.8 (2)
C11—C10—C9	121.6 (2)	C27—C26—C25	120.9 (3)
C11—C10—H10	119.2	C27—C26—H26	119.6
C9—C10—H10	119.2	C25—C26—H26	119.6
C10—C11—C6	122.0 (2)	C28—C27—C26	121.1 (4)
C10—C11—H11	119.0	C28—C27—H27	119.5
C6—C11—H11	119.0	C26—C27—H27	119.5
C9—N12—C13	118.9 (2)	C29—C28—C27	118.4 (3)
C9—N12—C14	118.8 (2)	C29—C28—H28	120.8
C13—N12—C14	115.9 (2)	C27—C28—H28	120.8
N12—C13—H13A	109.5	C28—C29—C30	121.9 (4)
N12—C13—H13B	109.5	C28—C29—H29	119.1
H13A—C13—H13B	109.5	C30—C29—H29	119.1
N12—C13—H13C	109.5	C29—C30—C25	120.0 (3)
H13A—C13—H13C	109.5	C29—C30—H30	120.0
H13B—C13—H13C	109.5	C25—C30—H30	120.0
N12—C14—H14A	109.5	N24—C31—C32	112.0 (3)
N12—C14—H14B	109.5	N24—C31—H31A	109.2
H14A—C14—H14B	109.5	C32—C31—H31A	109.2
N12—C14—H14C	109.5	N24—C31—H31B	109.2
H14A—C14—H14C	109.5	C32—C31—H31B	109.2
H14B—C14—H14C	109.5	H31A—C31—H31B	107.9
C16—C15—C20	116.8 (2)	C31—C32—C33	111.7 (3)
C16—C15—C5	122.3 (2)	C31—C32—H32A	109.3
C20—C15—C5	120.9 (2)	C33—C32—H32A	109.3
C17—C16—C15	121.8 (2)	C31—C32—H32B	109.3
C17—C16—H16	119.1	C33—C32—H32B	109.3
C15—C16—H16	119.1	H32A—C32—H32B	107.9
C16—C17—C18	121.3 (2)	C32—C33—H33A	109.5
C16—C17—H17	119.4	C32—C33—H33B	109.5
C18—C17—H17	119.4	H33A—C33—H33B	109.5
N21—C18—C19	122.1 (3)	C32—C33—H33C	109.5
N21—C18—C17	120.7 (3)	H33A—C33—H33C	109.5
C19—C18—C17	117.1 (2)	H33B—C33—H33C	109.5
			
C3—C1—C2—C3^i^	1.1 (4)	C5—C15—C16—C17	177.2 (2)
C4—C1—C2—C3^i^	−176.6 (2)	C15—C16—C17—C18	−0.9 (4)
C2—C1—C3—C2^i^	−1.1 (4)	C16—C17—C18—N21	−175.3 (3)
C4—C1—C3—C2^i^	176.7 (2)	C16—C17—C18—C19	2.1 (4)
C2—C1—C3—N24	179.6 (2)	N21—C18—C19—C20	176.4 (3)
C4—C1—C3—N24	−2.6 (4)	C17—C18—C19—C20	−1.0 (4)
C2—C1—C4—C5	−33.7 (4)	C18—C19—C20—C15	−1.6 (4)
C3—C1—C4—C5	148.6 (3)	C16—C15—C20—C19	2.8 (4)
C1—C4—C5—C6	−6.4 (4)	C5—C15—C20—C19	−176.0 (2)
C1—C4—C5—C15	177.1 (2)	C19—C18—N21—C22	8.5 (4)
C4—C5—C6—C7	131.4 (3)	C17—C18—N21—C22	−174.2 (3)
C15—C5—C6—C7	−52.0 (3)	C19—C18—N21—C23	159.1 (3)
C4—C5—C6—C11	−51.3 (3)	C17—C18—N21—C23	−23.6 (4)
C15—C5—C6—C11	125.4 (3)	C2^i^—C3—N24—C25	118.7 (3)
C11—C6—C7—C8	−0.1 (3)	C1—C3—N24—C25	−61.9 (4)
C5—C6—C7—C8	177.4 (2)	C2^i^—C3—N24—C31	−56.8 (3)
C6—C7—C8—C9	−1.9 (4)	C1—C3—N24—C31	122.5 (3)
C7—C8—C9—N12	−177.9 (2)	C3—N24—C25—C26	−11.8 (4)
C7—C8—C9—C10	2.9 (4)	C31—N24—C25—C26	163.6 (3)
N12—C9—C10—C11	178.9 (2)	C3—N24—C25—C30	169.8 (3)
C8—C9—C10—C11	−1.9 (4)	C31—N24—C25—C30	−14.8 (4)
C9—C10—C11—C6	−0.1 (4)	C30—C25—C26—C27	−0.3 (6)
C7—C6—C11—C10	1.2 (3)	N24—C25—C26—C27	−178.8 (4)
C5—C6—C11—C10	−176.3 (2)	C25—C26—C27—C28	1.1 (8)
C8—C9—N12—C13	15.2 (4)	C26—C27—C28—C29	−0.5 (9)
C10—C9—N12—C13	−165.6 (3)	C27—C28—C29—C30	−0.9 (9)
C8—C9—N12—C14	165.9 (3)	C28—C29—C30—C25	1.7 (7)
C10—C9—N12—C14	−14.9 (4)	C26—C25—C30—C29	−1.1 (5)
C4—C5—C15—C16	−55.0 (3)	N24—C25—C30—C29	177.4 (3)
C6—C5—C15—C16	128.2 (3)	C25—N24—C31—C32	98.4 (3)
C4—C5—C15—C20	123.8 (3)	C3—N24—C31—C32	−86.1 (3)
C6—C5—C15—C20	−53.0 (3)	N24—C31—C32—C33	−179.7 (3)
C20—C15—C16—C17	−1.6 (4)		

Symmetry codes: (i) −*x*+1/2, −*y*+3/2, −*z*+1.
